# Optimizing invitation letters for national cancer screening programs: Qualitative usability testing

**DOI:** 10.1016/j.pecinn.2026.100502

**Published:** 2026-09-03

**Authors:** Zeena Harakeh, Jessica Nieweg, Renske van Asten, Joris van Leenders, Marco Niënhaus, Pepijn van Empelen

**Affiliations:** a TNO, Netherlands Organization for Applied Scientific Research, Department of Child Health, Leiden, the Netherlands; b Markteffect, Market Research Agency, Eindhoven, the Netherlands; c National Health Screening Organization (Bevolkingsonderzoek Nederland – BVO NL), Utrecht, the Netherlands; d Department of Health, Medical and Neuropsychology, Leiden University, Leiden, Netherlands

**Keywords:** National cancer screening program, Qualitative usability testing, Co-creation, Communication materials, Health literacy

## Abstract

**Objectives:**

Participation in the Netherlands' three national cancer screening programs (breast, cervical, colorectal) is generally declining, especially among underserved populations. This study aimed to improve the official invitation letters distributed to eligible individuals by the National Health Screening Organization, in collaboration with the National Institute for Public Health and the Environment, thereby supporting informed decision-making about participation in the national screening programs. Two revised invitation letters were developed based on recommendation derived from previous research, aimed at improving design and communication.

**Methods:**

Usability testing was conducted among 27 Dutch adults (aged 30–75): approximately two-thirds of whom had a lower level of education, lower socioeconomic status, or a migration background. Participants reviewed the two revised invitation letters either online or in person and provided feedback through semi-structured interviews and using the think-aloud method. Responses were qualitatively analyzed, using the framework method, cluster analysis and mind mapping, to identify and structure key themes.

**Results:**

Participants found the revised letters clearer, more concise, and better structured than the original letters. Bolded key information and accessible contact details were appreciated and evaluated as means to ease access and comprehension. Icons generally did not improve the understandability of the letters and were considered unnecessary. Additionally, participants suggested adopting a more personal tone and adding information about the benefits of screening.

**Conclusion:**

Our findings show that co-creating revised screening invitation letters with end users improves clarity and accessibility, supporting informed decision-making and more inclusive public health communication.

**Innovation:**

This research is innovative by co-creating cancer screening invitation letters with end-users, especially those among underserved groups, and led to several participant-driven improvements which will be adopted in the national population-based cancer screening program.

## Highlights

• Co-creation with users contributes to inclusivity and relevance of health communication letters.
• Clarity, structure, and user-friendliness were significantly improved.
• Bolded text and clear contact support information were positively evaluated.
• Icons caused confusion and were excluded from final design.
• Organizational health literacy is essential.

## 1 Introduction

### 1.1 Background

Cancer is a major health problem. Population-based screening contributes to the early detection of cancer, reduces mortality rates, and improves survival outcomes [1], [2], [3]. In the Netherlands, three nationwide, cost-free population-based cancer screening programs are offered for breast, cervical and colorectal cancer. In 2023, approximately 2.6 million people participated in these programs [4]. Screening is offered every two years for breast cancer (women aged 50–75 years), every five or ten years for cervical cancer (women aged 30–60 years), and every two years for colorectal cancer (men and women aged 55–75 years). The programs are centrally organized by the National Institute for Public Health and the Environment (“Rijksinstituut voor Volksgezondheid en Milieu” also referred to as “RIVM”) in collaboration with the National Health Screening Organization (“Bevolkingsonderzoek Nederland” also referred to as “BVO NL”). Communication takes place by post at three key moments: the invitation letter (including a brochure and, where applicable, a self-test), a reminder letter (sometimes including an additional self-test), and the test result letter. Eligible individuals are identified through population registries and receive invitation letters to support informed decision-making about participation. Participation is voluntary and does not depend on referral by a healthcare professional. Despite the benefits of screening, participation rates have generally declined in recent years. In 2024, participation rates were 54.4% for cervical cancer screening, 67.1% for colorectal cancer screening, and 65.3% for breast cancer screening; compared with 2023, participation increased slightly for cervical and colorectal cancer screening, whereas breast cancer screening continued to decline [5]. Participation is particularly lower among underserved populations, including those with a lower socioeconomic status and a non-Western migration background [6]. One possible explanation is communication barriers, such as language difficulties and limited health literacy [7]. Screening information is often complex, and approximately one-third of the Dutch population has limited health literacy skills, making it more difficult to access, understand, and use health information when deciding whether to participate in screening [8], [9].

Previous co-design research with eligible participants – particularly among underserved populations (i.e. older adults, people with low literacy, and individuals with a migration background) – demonstrated that the accessibility and clarity of invitation letters for screening programs can be significantly improved [10]. Key recommendations included a) using accessible and understandable language, b) reducing text volume, and c) emphasizing key words in bold to improve readability. Additionally, it was found that a) use of pictograms and icons could help to improve the structure of the letter, b) a language guide at the beginning of the letter to explain its content in multiple languages would contribute to understandability of the information, c) the possibility for telephone contact is essential especially for people with low health literacy, d) a short web address could guide users to the webpage with more information about the screening programs, and e) a QR code—clearly explained with a brief description of its purpose and how to use it—could make it easier for users to directly access the relevant webpage. Additional research among individuals with lived experience of low literacy confirmed these findings and highlighted the need for QR-code scanning instructions, a more prominent Client Services phone number, and a QR code within the contact block [11]. Several of these recommendations were subsequently implemented by BVO NL, in collaboration with RIVM, in revised versions of the letters to the clients (e.g., the invitation letter and the test result letter). These revisions targeted three levels of communication: macrostructure (organization of information; i.e. short paragraphs, a contact information block), microstructure (clarity and coherence of text; i.e. shorter and simplified text), and visual language (use of visual elements to support understanding; i.e. bolded keywords, QR code linking to more detailed information, icons to structure content, and language-support features such as flag icons) [12], [13].

### 1.2 The present study

**Fig. 1:**
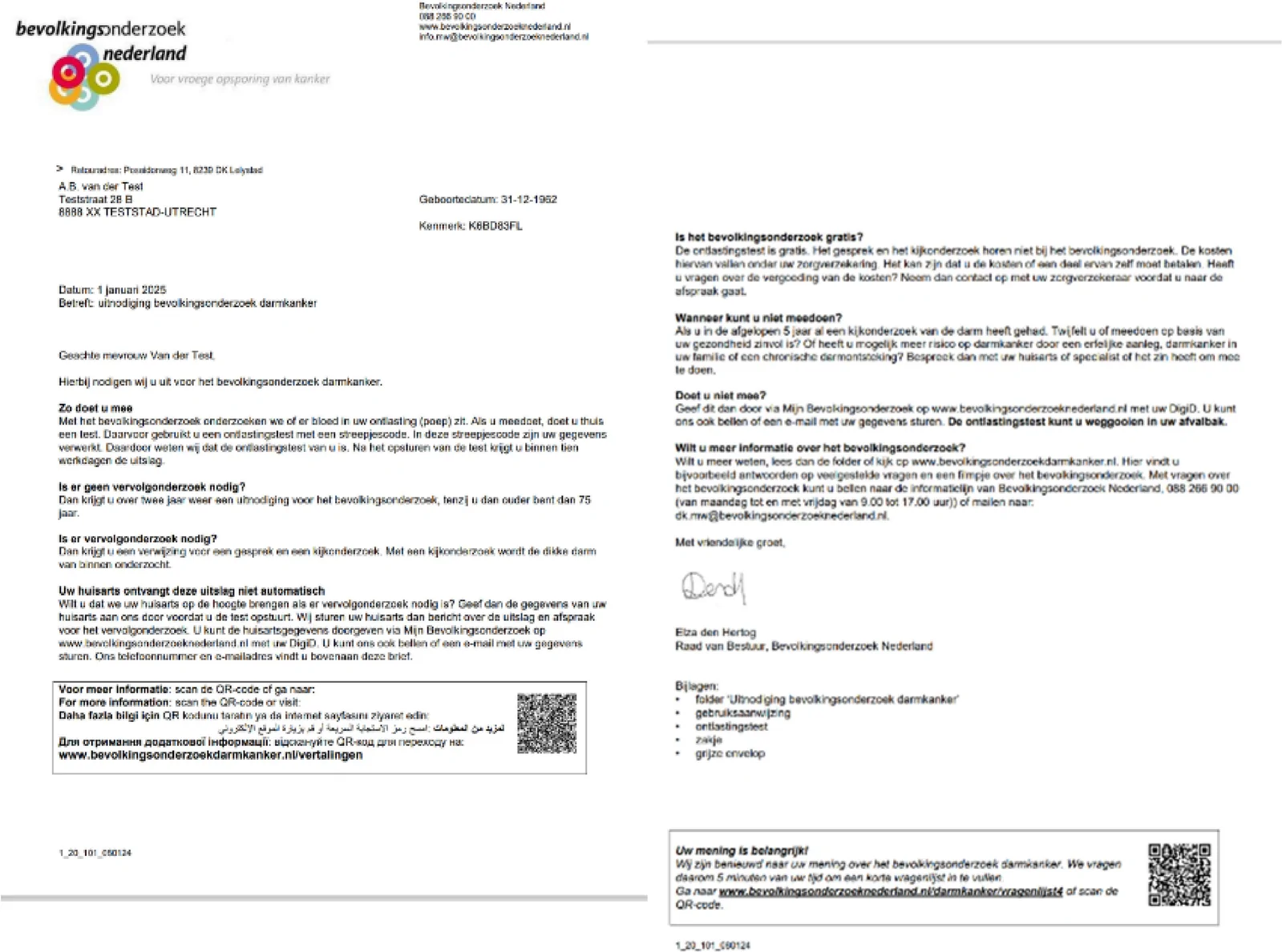
The original invitation letter for one of the three population cancer screening programs (i.e. colorectal cancer)**.**

The present study aimed to improve the clarity, acceptability and user-friendliness of the invitation letters for the national population based cancer screening programs before their nationwide implementation. The original invitation letter is depicted in Fig. 1. Two revised versions of the invitation letter were developed based on previous research [10], [11], focusing on design and communication enhancements. The goal of the present study was to examine whether the invitation letters of the national cancer screening program (breast, cervical and colorectal cancer) contributed to text comprehension and usability of the letter, especially among underserved populations (i.e. individuals with lower level of education or socioeconomic status, and/or with a migration background).

## 2 Methods

### 2.1 Participants

Twenty-seven Dutch individuals aged 30 to 75 who were eligible for one of the Dutch national cancer screening programs participated in the study. There are different invitational letters for each screening program: i.e. breast, cervical and colorectal cancer. Each invitational letter was evaluated by a subgroup of nine participants. The participants in this study were recruited through the panel of Markteffect (i.e. a Dutch market research agency), which is an ISO certified panel and consists of over 100,000 panel members spread across the entire 18+ population. To ensure adequate representation of people with a lower education level, lower socioeconomic status (SES) and/or a migration background, two-thirds of participants met at least one of these characteristics. We took this into account in the recruitment by categorizing the participants based on SES, ethnicity, and digital skills. SES was determined using participants' educational level, housing situation (owning or renting), and income.

### 2.2 Procedure

**Fig. 2:**
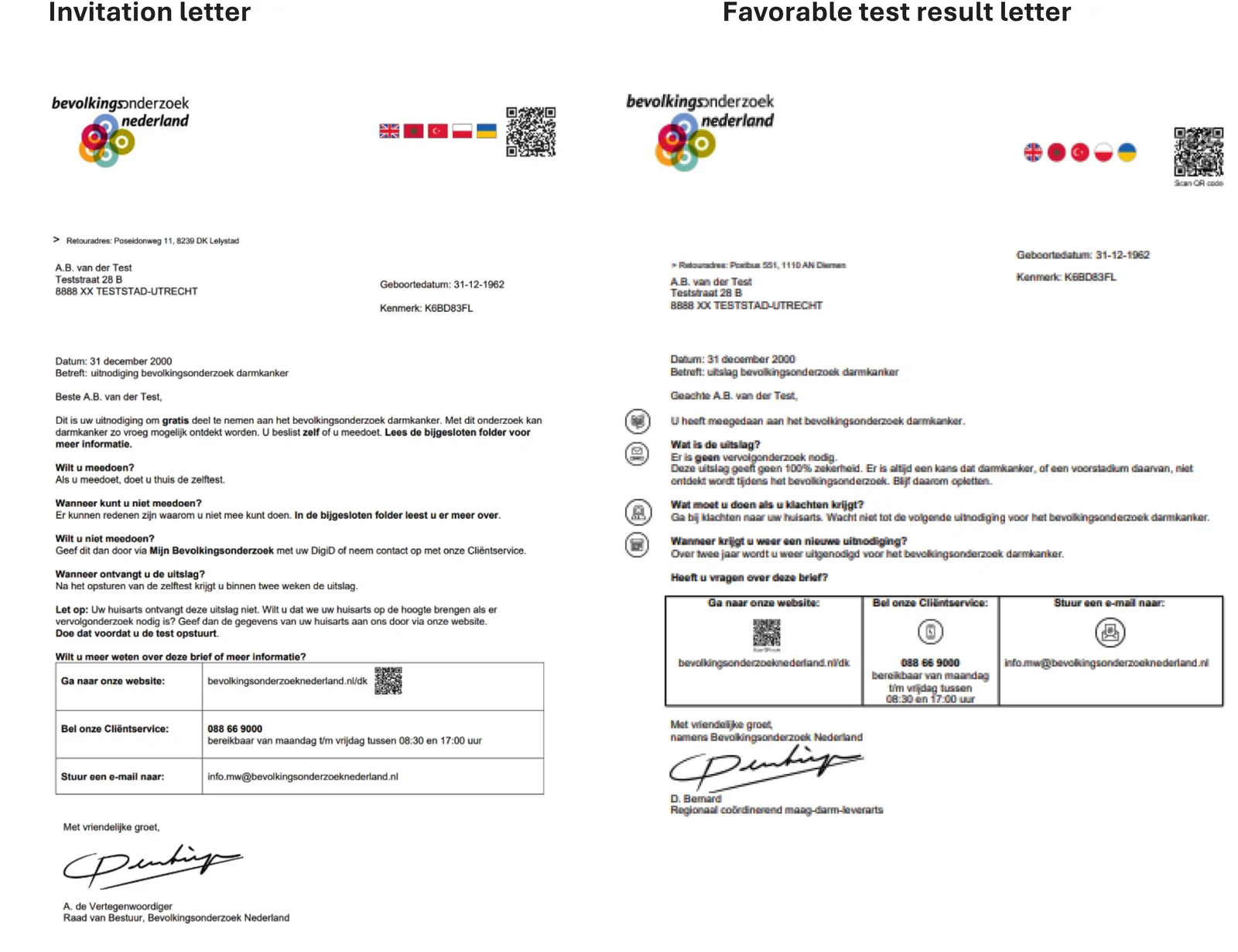
Revised invitation and favorable test result letters aligned with a client journey in the colorectal cancer screening program. *Note.* The format of the invitation letter and favorable test result letter varied between the three cancer screening programs (breast, cervical and colorectal cancer). Nevertheless, all participants evaluated and compared the two revised versions of the national cancer screening letters.

This study is part of a broader research project approved by the TNO ethical review committee for non-medical human research (approval number #2023–013). It aimed to evaluate and compare two revised versions of the national cancer screening invitation letter through usability testing via interviews. We used a client journey approach, in which each participant was first presented with one of the two revised invitation letters, followed by a favorable test result letter (including the revisions of the other version of the invitation letter), and finally with the original letter. These two revised letters varied across several design and communication elements (i.e. QR code, language icon flags, icons, positioning of contact details and salutation) (see Table 1). Participants were presented at random order one of the two versions of the revised invitation letter, and followed briefly by the revised favorable test result letter (see Fig. 2). The variations in these two revised versions were systematically compared within the study.

**Table 1:** Comparisons between the differences in the two versions of the revised letter presented to participants and the version of the current letter.

	Differences in the two versions	Original (current letter)
**Length**	Both short	Long
**QR code**	Yes vs Yes, with an explanatory note	No
**Language Icon flags**	Yes, rectangles vs Yes, circles	No
**Bolded words**	Both yes	No
**Icons**	No vs Yes	No
**Positioning of Contact details**	Contact block, horizontal placement vs Contact block, vertical placement	In text
**Salutation**	Formal vs More personal	Formal

The interviews were conducted with the participants via MS Teams or in person at a location in Eindhoven. Each interview lasted on average between 45 and 60 min. Data collection took place from 11 to 28 March 2025. Participants gave informed consent via questionnaire (including permission for recording, the presence of a third party, and anonymized reporting), then reconfirmed it by phone and email. Two researchers were present during each interview. One researcher conducted the interview and was visible to the participant, while the other researcher took notes and remained off camera. Interviews explored three topics: a) preferences regarding design and communication elements, b) ease of reading, and c) whether the letter helped participants understand the screening program and how to participate. The participants could provide their responses via the think-aloud method [14]. At the end of the interview, they also were shown the original letter and asked what they appreciated about the existing letter compared to the revised versions, and what they considered improvements in the new versions. Participants received a financial incentive (€40 gift voucher) for participating in the interview.

### 2.3 Data analyses

Audio recordings were transcribed using Markteffect's in-house software based on OpenAI's Whisper Large V2 model. The transcripts were then analyzed using the framework method [15] to identify key concepts through systematic grouping of data. These concepts were further examined using cluster analysis to derive overarching themes [16]. Similarities and differences were also examined between the three cancer screening programs (breast, cervical and colorectal cancer) and between participant characteristics (e.g., education level). Findings were synthesized using mind mapping to structure key themes (core message, call to action, and guidance), similarities and differences, from which conclusions and recommendations were drawn [17]. We applied the Pyramid principle [18] to structure our findings from the most important insights to the less critical ones. Subsequently, the retrieved themes were categorized into three levels of text cohesion: macrostructure, microstructure and visual language/aids [12], [13], [19].

## 3 Results

### 3.1 Sample characteristics

Most participants were female, aged over 50 years (*M* = 58.4 years; *SD* = 10.8 years) and of Dutch nationality (see Table 2). The majority of the participants were female because women are invited to participate in all three screening programs, while men are only invited for one (the colorectal cancer screening program). Furthermore, four of the 27 participants reported not participating in the national cancer screening programs.

**Table 2:** Demographic characteristics of the sample.

Category		Count (*n*)
Gender		
	Male	4
	Female	23
Age		
	30–39 years	3
	40–49 years	2
	50–59 years	9
	60–69 years	8
	70–75 years	5
Education Level^1^		
	Low	5
	Medium	10
	High	12
Ethnicity^2^		
	Dutch	24
	Surinamese	2
	Turkish	1
Socioeconomic Status^3^		
	Low	4
	Medium/High	23

Note.

1 Operationalization education level according to Statistics Netherlands [20].

2 If the participant or one of their parents has a non-Dutch nationality, they are categorized according to the nationality of the participant or their parent.

3 Socioeconomic status (SES) is determined based on educational level, housing situation (ownership or rental), and income. A participant with a low SES is characterized by a low educational level, a monthly income below €2600, and rental housing.

### 3.2 Overall evaluation of the revised letter

**Fig. 3:**
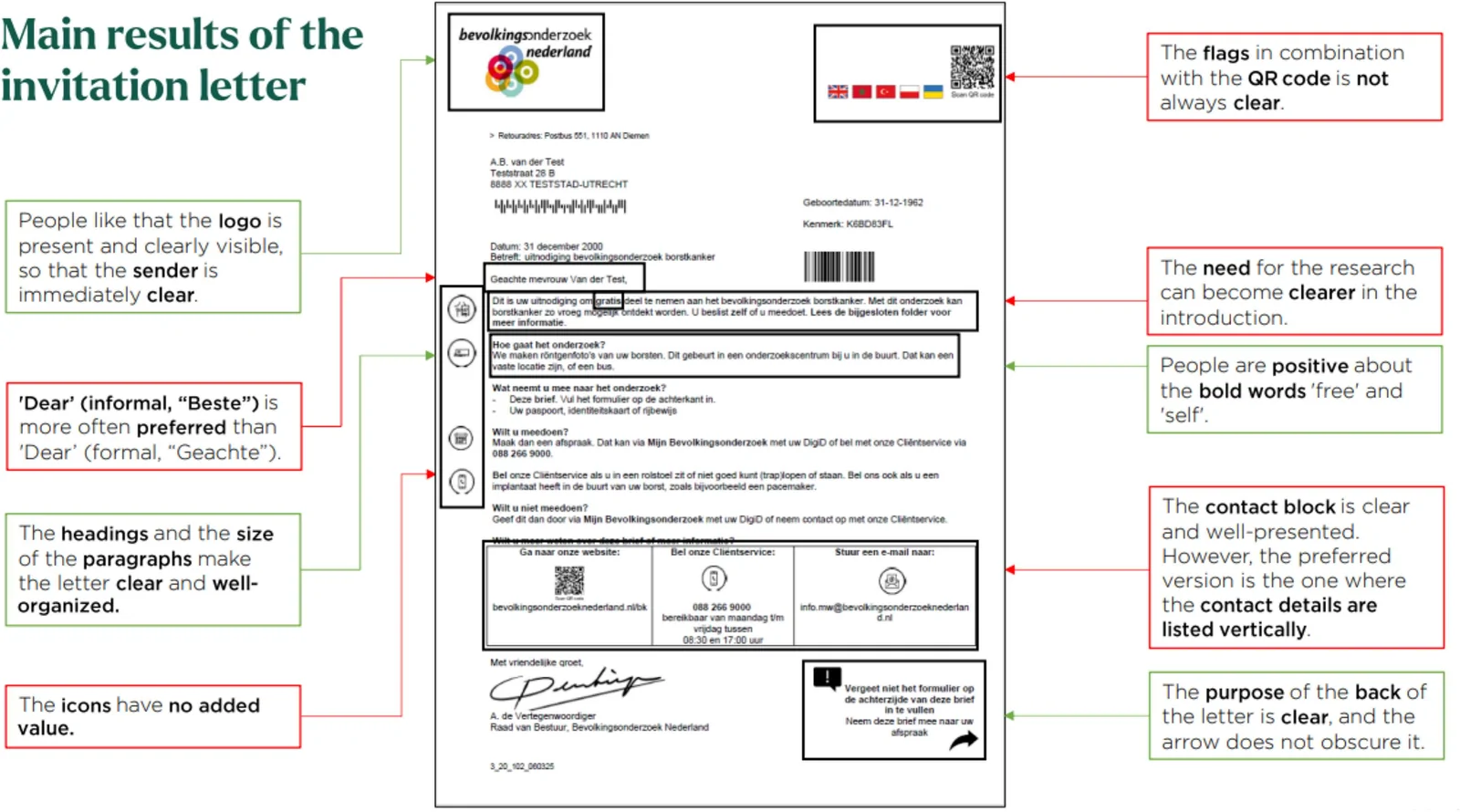
Results regarding what participants thought of the invitation letter. *Note.* The rectangles in green are positive points, and in red are suggestions for improvement. (For interpretation of the references to color in this figure legend, the reader is referred to the web version of this article.)

The new revised invitation letters were received positively and seen as an improvement over the original ones. The revised invitation letter was perceived as clear, well-structured, complete, and the purpose of the letter and the screening was evident. Participants acknowledged several strengths of the revised letter, as well as some areas for improvement (see Fig. 3). We categorized the results into three categories: 1) the macrostructure, 2) the microstructure and 3) the use of visual language/aids.

#### 3.2.1 Macrostructure

The macrostructure refers to the overall organization, layout, and structuring of the text. The results are depicted in Table 3. The following aspect was mentioned as a strength of the letter: the letter clearly indicated which screening program the person is being invited to (breast cancer/colorectal cancer/cervical cancer screening) —helpful for those participants who are invited to multiple screenings at once. The contact information layout depicted in the letter was mentioned as an area for improvement. The contact details were included in the letter but participants indicated that the contact block arranged vertically has their preference. The majority preferred the contact block to be formatted with both rows and columns, as this reads more naturally. *“Remove the contact block from the text, I would add some blank lines in between.”*

**Table 3:** Features (macrostructure, microstructure and visual language/aids) of the invitation letter: Strengths and areas for improvement.

Feature	Strength	Quotations	Areas for Improvement	Quotations
**Macrostructural features**				
**Screening program identification**	Specifies which screening the invitation concerns, aiding clarity.	“*When you open it [the letter], you see what it is intended for.”*	–	
**Contact information layout**	Contact details are included.		Vertically arranged contact block with spacing is preferred for readability.	*“You're reading, aren't you? From top to bottom. So why should you start reading from left to right now?”*
*Microstructural Features*				
**Language clarity**	The letter is generally easy to understand.	*“It is clear that it is from Bevolkingsonderzoek Nederland and it is well organized. It's an A4 sheet and the layout seems easy to read.”*	Concerns about accessibility for people with a migration background or low literacy.	*“A functionally illiterate person doesn't read at all. Then you have a group that is close to illiteracy. They also have trouble with it.”*
**Completeness of content**	No essential information is perceived as missing.	*“Certain important things are in bold. I would read he brochure, and if I still had questions, I would call that 088 number.”*	Some practical questions (e.g., deadlines, response times) could be addressed directly in the letter.	*“Is it mandatory to unsubscribe if you don't want to participate? […] I would add something like: ‘There may be reasons why you cannot participate. We would appreciate it if you let us know.’ That sounds a bit friendlier.”* *“If you send an email, how long does it take to get a response?*” “*Within what timeframe do you have to return [the colorectal cancer self-test]? […] If there is a deadline, I would definitely include that*.”
**Tone of voice**	The tone is generally appropriate and respectful.	*“It's just clear and businesslike. Yes, what I expect from such a letter.”*	Some participants prefer a more personal tone, especially in the results letter.	*The tone could perhaps be a little more personal, a little friendlier. It's so standard.”*
**Motivation to participate**	The brochure includes rationale for participation.	*“Otherwise, you have to expand completely with how the research takes place. So I find it more convenient if it is in the enclosed folder.”*	*The letter lacks a clear explanation of why participation is important; urgency is missing.*	*“You can participate, but it is not really clear that it is about your health. That's why it's important to introduce why it's important.”*
**Visual language/aid Features**				
**Use of bold text**	Bolded keywords help clarify the message and support skimming.	*“What I think is good is that some things are indeed bold. That it is free also makes it more plausible to do it.”*	Some participants would prefer more keywords to be bolded for emphasis.	*“For example, I would choose to do ‘those symptoms’ in bold instead of the words ‘go to your doctor’.”*
**Logo placement**	Clearly identifies the sender at first glance.	“*Above the heading, then you immediately know that the logo is from het Bevolkingsonderzoek. I think that's very clear.”*	–	
**Use of icons**	–		Icons are unclear, cause confusion, and clutter the layout.	*“Rather without, because I have no idea what those icons mean. Without icons, I don't have to wonder why they are there.”*
**Translation accessibility**	Translation block is present.		QR code lacks clarity; rectangular flags at the top are preferred for realism and visibility.	*“Those flags. That's a bit unclear with that QR-code next to it.”*

#### 3.2.2 Microstructure

The microstructure refers to the clarity, coherence, and linguistic features at the word and sentence level. The results are depicted in Table 3. Participants mentioned various strengths of the revised letter. First, participants found the letter and its message easy to understand. A few participants wondered whether it is also understandable for people with a migration background or those who have difficulty reading. Second, participants also did not feel that any information is missing. Answers to any questions the letter may raise could be found in the brochure, which they consider the best place for additional information. No extra information needed to be added to the letter, as this keeps it short and clear. If they still had questions after reading the leaflet, they would visit the website or call client services.

In addition, some areas for improvement were mentioned by participants. First, the tone of voice of the letter is good. Most participants feel addressed by the letter, but some would prefer it to be more personal—especially the results letter. For example, it was suggested to add “how nice” to the sentence “No further investigation is needed” and to use “dear” instead of “esteemed.” Those who preferred “esteemed” did so mainly because it sounded more formal, which they felt is more appropriate for a letter from BVO NL. Second, although most participants participated in the population screening themselves, some felt the letter did not sufficiently explain why participation is important. They missed a sense of urgency. While this explanation was included in the brochure, participants considered it too essential to be mentioned only there. They wanted the letter itself to clearly state why participation is valuable.

#### 3.2.3 Visual language/aids

Visual language/aids concerns the use of visual elements (e.g., images, color and typography) to support and enhance the meaning of the text. The results are depicted in Table 2. The following aspects were mentioned as strengths of the letter. First, the bolded words in the letter helped make the purpose of the letter clear. Since participants tended to skim-read, a brief and well-organized letter with bolded key terms helped convey the message clearly. Participants were positive about the use of bold text—especially for words like “free of charge,” “you decide whether to participate,” and “no further testing needed.” Some would prefer even more key words to be bolded to emphasize their importance. Second, the logo in the top right corner of the letter immediately showed who the sender of the letter is from.

There were also some areas for improvement mentioned. First, it was not clear to everyone what the icons exactly meant, they caused confusion and did not contribute to the message of the letter, and made the letter feel cluttered. Second, with regard to the text block for translation of the letter, participants preferred (rectangular) flag icons with a clear link to a QR code. Some participants spontaneously mentioned preferring rectangular flags because they looked more realistic. However, it was not clear to everyone that the QR code needed to be scanned for translation. Participants wanted a clearer reference of this. Furthermore, participants preferred the flags at the top of the letter. This was clearer than the current text block for translations.

## 4 Discussion and conclusion

### 4.1 Discussion

**Fig. 4:**
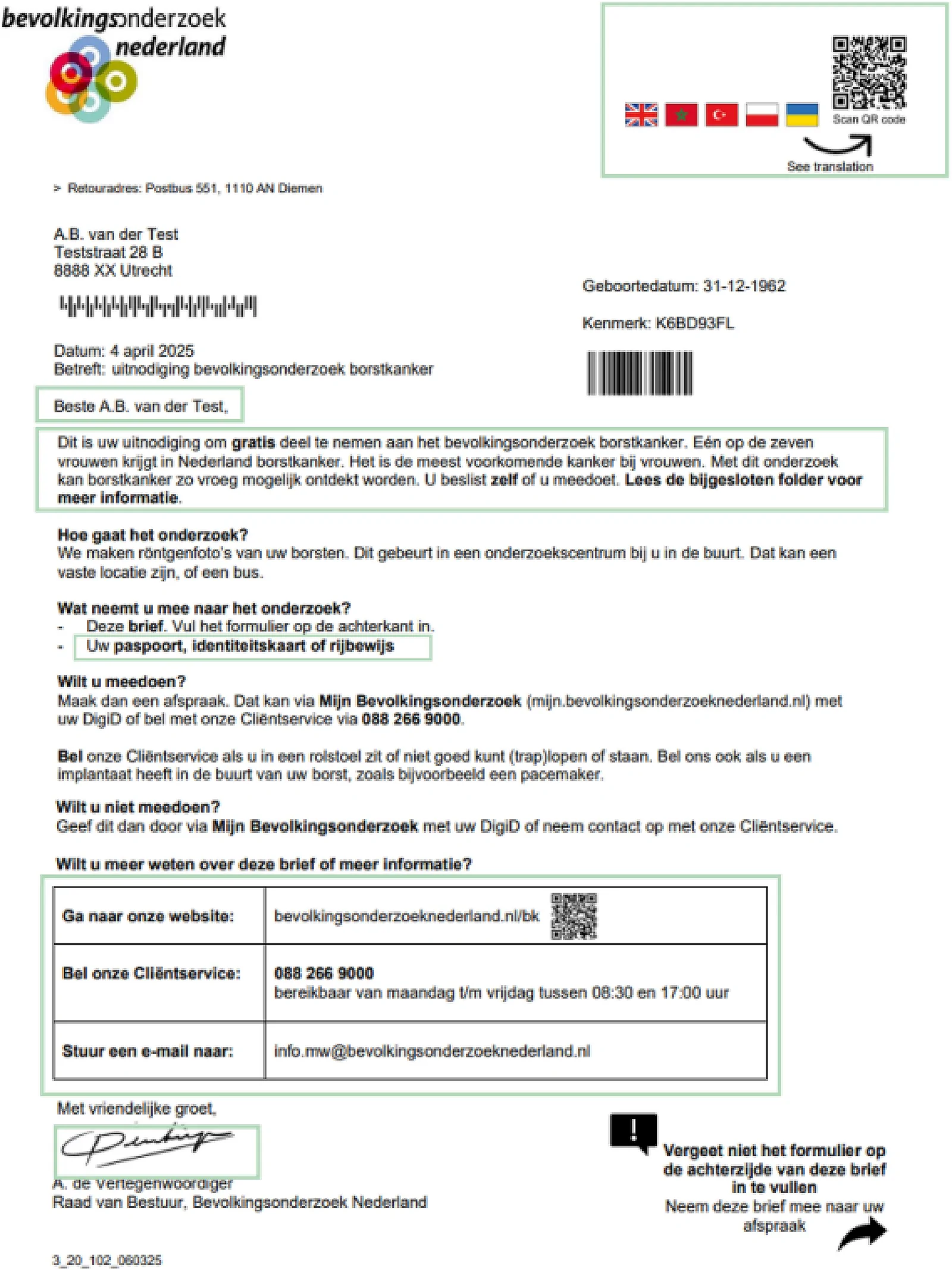
The optimized and implemented invitation letter on the basis of the results of the study.

The aim of this study was to evaluate whether revisions of the cancer screening communication materials could help to improve acceptance and user-friendliness, prior to nationwide implementation. Overall, the revised invitation letters were perceived as an improvement over the original version. Participants described the revised letters as clearer, better structured, and more complete, with the purpose of both the letter and the screening program being readily understandable. To analyze these findings, we categorized the results into three levels: macrostructure (overall organization and layout), microstructure (linguistic clarity and coherence), and visual language/aids (use of formatting and visual elements to support meaning). Strengths were identified across all three levels. These included greater clarity (including clarity of which screening program it concerns), more complete content, an appropriate and respectful tone of voice, and effective visual emphasis through bold text and logo placement. There were also some areas for improvement that were noted by the participants, such as the layout of contact information and the clarity of icons and translation references. The study findings have led to final adaptations of the invitation letter for the national cancer screening programs, which will be implemented nationwide (see Fig. 4). Furthermore, the study results underscore the importance of tailoring communication to the needs of diverse user groups and demonstrate how co-creation can enhance the effectiveness and inclusivity of public health messaging.

Our findings confirm that simplifying complex health communication—by reducing text and emphasizing clarity—can enhance comprehension without compromising the legal or medical-scientific accuracy of the information. Participants appreciated the concise format of the revised letters, supporting the principle of “less is more.” This is in line with previous studies which suggested that brief communication may improve decision-making and reduce cognitive burden, especially in risk communication [21]. Also, the tone of voice and visual emphasis such as use of bold text and logo placement can contribute to a better understanding and enhance an informed choice [21]. Furthermore, the layout of contact information and the translation references were improved in the final optimized letter (see Fig. 4). However, the use of icons in the revised letters led to mixed responses and thus, were not included in the final optimized letter. While intended to support structure and comprehension, several participants found them confusing or distracting. This suggests that icons should be carefully tested for interpretability, relevance and standardization. Their effectiveness may also depend on the context (e.g., app, online platform, hospital environment) in which they are used [13]. Ambiguity in icon meaning may hinder rather than help understanding if they are implemented poorly [13].

Our findings can contribute to organizational health literacy (i.e. the ability of institutions to make health information accessible). Health literacy is often defined as an individual trait. However, it is not solely determined by a person's skills; it also depends on the complexity and demands of the surrounding context and environment. Organizations play a crucial role in shaping this context. By ensuring that communication is accessible and understandable to all —regardless of health literacy levels — organizations can promote equitable access to health information, prevention, and care, and help to reduce health disparities [22], [23]. However, beyond accessibility and comprehensibility, the concept of action capability is also essential for understanding participation in population screening programs. Even when information is clear and accessible, other barriers—such as emotional readiness, cultural beliefs, or logistical constraints—may prevent individuals from acting. Addressing these barriers requires a holistic approach that goes beyond written communication and considers the broader context of people's lives [e.g., [24]].

In our study we applied a user-centered design, which is important when developing and improving (inclusivity of) public health messaging and communication. It is important to involve end-users early in the development process to understand their needs and their interaction with communication materials to identify the strengths and weaknesses [25]. Thus, the benefits of co-creation and involving end-users in prototype testing were evident. Moreover, co-creation fosters inclusivity [10] and ensures that communication materials resonate with diverse audiences, including underserved populations.

#### 4.1.1 Limitations

This study has a few limitations that should be considered when interpreting the findings. First, the sample size was relatively small (*N* = 27), which may limit generalizability of the findings. However, data saturation was reached during the interviews, indicating that the range of perspectives was sufficiently captured for the purpose of usability testing and qualitative analysis. Second, most participants were female. This intentional imbalance is explained by the fact that women are invited to participate in all three national cancer screening programs (breast, cervical, and colorectal cancer), whereas men are only invited for one program (colorectal cancer). As such, the gender distribution reflects the structure of the screening programs and the target population. Nevertheless, future studies may benefit from including a more balanced sample to explore potential gender-specific preferences in communication materials. Third, only a limited number of participants with a migration background were included, restricting the extent to which the findings reflect the diversity of cultural and linguistic perspectives within the screening population. Future studies should include more diverse participant groups and larger samples to explore possible differences in communication needs and preferences between population subgroups. Finally, although potential differences in feedback according to socioeconomic status and migration background were explored, no meaningful differences emerged between these groups. Other potentially relevant characteristics, such as health literacy, previous screening experience, and digital skills, were not examined and may influence how screening communication is perceived and used. Future research should investigate whether communication needs and preferences vary across these characteristics to further support tailored and inclusive screening communication.

### 4.2 Innovation

This study is innovative in both its approach and its practical implications for public health communication within the Dutch national cancer screening program. Previous studies have highlighted the importance of user-centered design, co-creation, and health literacy principles in the development of health communication materials and decision support tools [22], [25], [26], [27]. Building on this work, the present study actively involved end-users—including individuals with lower socioeconomic status, limited health literacy, or a migration background—in the redesign of national cancer screening invitation letters. Unlike traditional top-down communication strategies, this approach incorporated end-user perspectives to improve the language, structure, and visual presentation of the materials. In addition, usability testing using the think-aloud method provided detailed insights into how intended recipients understood and interpreted specific communication features. A key innovation of this study is that the findings directly informed invitation letters that were subsequently implemented within the Dutch national cancer screening program. This demonstrates how engagement with intended audience can strengthen organizational health literacy and improve alignment between public health communication and the needs of diverse populations [22], [23]. Whereas many studies remain limited to the development and evaluation of prototypes, the present study translated user-informed improvements into nationwide practice, illustrating the potential of user-centered approaches to support informed decision-making and reduce health disparities*.*

## 5 Conclusion

This study demonstrates that revising health communication materials—specifically invitation letters for national cancer screening programs—while actively involving end-users through co-creation, can significantly enhance clarity, accessibility, and user-friendliness. By simplifying the text, emphasizing key information, and tailoring the design to diverse needs, the revised letters support informed decision-making and contribute to more inclusive public health communication that is suitable for nationwide implementation.

## CRediT authorship contribution statement

**Zeena Harakeh:** Writing – review & editing, Writing – original draft, Funding acquisition, Conceptualization. **Jessica Nieweg:** Writing – review & editing, Methodology, Investigation. **Renske van Asten:** Methodology, Investigation. **Joris van Leenders:** Writing – review & editing, Methodology, Investigation. **Marco Niënhaus:** Writing – review & editing, Methodology, Conceptualization. **Pepijn van Empelen:** Writing – review & editing, Funding acquisition, Conceptualization.

## Ethics declaration

Written informed consent to take part in the study and to publish the article has been obtained from all participants or their legal representatives. The privacy rights of participants have been observed.

This study was performed in compliance with relevant laws, regulatory frameworks and guidelines where the research took place.

This study was approved by the TNO ethical review committee for non-medical human research.

(Approval No. #2023–013)

## Declaration of generative AI and AI-assisted technologies in the writing process

During the preparation of this work the author(s) used M365 Copilot in order to shorten and streamline the manuscript. After using this tool/service, the author(s) reviewed and edited the content as needed and take(s) full responsibility for the content of the published article.

## Funding statement

This work was funded by 10.13039/501100001826ZonMw (grant number 10690462410008). The data collection was carried out by Markteffect – an independent Dutch market research agency - on behalf of National Health Screening Organization (BVO NL), and was funded by the Ministry of Health, Welfare and Sport (VWS) and part of a greater project involving the improvement of the accessibility of the National cancer screening programs in the Netherlands, especially for people with low health literacy.

## Declaration of competing interest

The authors declare no conflicts of interest.

## Figures and Tables

**Table:** 

Title and Abstract	
**Title** - Concise description of the nature and topic of the study Identifying the study as qualitative or indicating the approach (e.g., ethnography, grounded theory) or data collection methods (e.g., interview, focus group) is recommended	p. 1
**Abstract** - Summary of key elements of the study using the abstract format of the intended publication; typically includes background, purpose, methods, results, and conclusions	p. 2
**Introduction**	
**Problem formulation** - Description and significance of the problem/phenomenon studied; review of relevant theory and empirical work; problem statement	p. 3–5
**Purpose or research questio**n - Purpose of the study and specific objectives or questions	p. 5
**Methods**	
**Qualitative approach and research paradigm** - Qualitative approach (e.g., ethnography, grounded theory, case study, phenomenology, narrative research) and guiding theory if appropriate; identifying the research paradigm (e.g., postpositivist, constructivist/ interpretivist) is also recommended; rationale**	p. 6
**Researcher characteristics and reflexivity** - Researchers' characteristics that may influence the research, including personal attributes, qualifications/experience, relationship with participants, assumptions, and/or presuppositions; potential or actual interaction between researchers' characteristics and the research questions, approach, methods, results, and/or transferability	*not applicable*
**Context** - Setting/site and salient contextual factors; rationale**	p. 6
**Sampling strategy** - How and why research participants, documents, or events were selected; criteria for deciding when no further sampling was necessary (e.g., sampling saturation); rationale**	p. 5, p. 11
**Ethical issues pertaining to human subjects** - Documentation of approval by an appropriate ethics review board and participant consent, or explanation for lack thereof; other confidentiality and data security issues	p.5–6
**Data collection methods** - Types of data collected; details of data collection procedures including (as appropriate) start and stop dates of data collection and analysis, iterative process, triangulation of sources/methods, and modification of procedures in response to evolving study findings; rationale**	p. 6
**Data collection instruments and technologies** - Description of instruments (e.g., interview guides, questionnaires) and devices (e.g., audio recorders) used for data collection; if/how the instrument(s) changed over the course of the study	p. 6
**Units of study** - Number and relevant characteristics of participants, documents, or events included in the study; level of participation (could be reported in results)	p. 5, Table 2
**Data processing** - Methods for processing data prior to and during analysis, including transcription, data entry, data management and security, verification of data integrity, data coding, and anonymization/de-identification of excerpts	p. 6–7
**Data analysis** - Process by which inferences, themes, etc., were identified and developed, including the researchers involved in data analysis; usually references a specific paradigm or approach; rationale**	p. 6–7
**Techniques to enhance trustworthiness** - Techniques to enhance trustworthiness and credibility of data analysis (e.g., member checking, audit trail, triangulation); rationale**	*not applicable*
**Results/findings**	
**Synthesis and interpretation** - Main findings (e.g., interpretations, inferences, and themes); might include development of a theory or model, or integration with prior research or theory	p. 7–9
**Links to empirical data** - Evidence (e.g., quotes, field notes, text excerpts, photographs) to substantiate analytic findings	p. 7–9. Table 3
**Discussion**	
**Integration with prior work, implications, transferability, and contribution(s) to the field -** Short summary of main findings; explanation of how findings and conclusions connect to, support, elaborate on, or challenge conclusions of earlier scholarship; discussion of scope of application/generalizability; identification of unique contribution(s) to scholarship in a discipline or field	p. 9–11
**Limitations** - Trustworthiness and limitations of findings	p. 11
**Other**	
**Conflicts of interest** - Potential sources of influence or perceived influence on study conduct and conclusions; how these were managed	p. 12
**Funding** - Sources of funding and other support; role of funders in data collection, interpretation, and reporting	p. 12

## Data Availability

The data sets generated and analyzed during this study are available from the National Health Screening Organization (BVO NL) upon reasonable request.

## Glossary

Population-based screening: Systematic testing offered to a defined group (e.g., by age or gender) to detect disease early, often before symptoms appear.
Socioeconomic status (SES): An individual's social and economic position, often measured by educational level, income, and type of occupation.
Health literacy: The ability of individuals to access, understand, and apply health information to make informed decisions.
Invitation letter: Official communication sent to eligible individuals, inviting them to participate in a screening program.
Co-creation: Collaborative process involving end-users (such as patients or citizens) in the design and development of communication materials or interventions.
Usability testing: Evaluation method where real users review and provide feedback on materials or products to assess clarity, accessibility, and effectiveness.
Macrostructure: The overall organization and logical flow of a text or document.
Microstructure: The clarity and coherence of language at word and sentence level within a document.
Visual language/aids: Use of visual elements (e.g., icons, bold text, QR codes) to enhance understanding and accessibility of information.
Think-aloud method: A qualitative research technique where participants verbalize their thoughts while completing a task, used to gain insight into their reasoning and understanding.
